# Calcium Phosphate Incorporated Bacterial Cellulose-Polyvinylpyrrolidone Based Hydrogel Scaffold: Structural Property and Cell Viability Study for Bone Regeneration Application

**DOI:** 10.3390/polym11111821

**Published:** 2019-11-06

**Authors:** Probal Basu, Nabanita Saha, Radostina Alexandrova, Petr Saha

**Affiliations:** 1 Centre of Polymer Systems, University Institute, Tomas Bata University in Zlin, 760 01 Zlín, Czech Republic; probal@utb.cz (P.B.); saha@utb.cz (P.S.); 2 Institute of Experimental Morphology, Pathology and Anthropology with Museum, Bulgarian Academy of Sciences, 1113 Sofia, Bulgaria; rialexandrova@hotmail.com

**Keywords:** bacterial cellulose, calcium phosphate, hydrogel scaffolds, degradation, mechanical property, bone regeneration

## Abstract

This work focuses on the analysis of structural and functional properties of calcium phosphate (CaP) incorporated bacterial cellulose (BC)-polyvinylpyrrolidone (PVP) based hydrogel scaffolds referred to as “CaP/BC-PVP”. CaP is incorporated in the scaffolds in the form of hydroxyapatite (HA) and β-tricalcium phosphate (β-TCP) in different concentrations (β-TCP: HA (*w*/*w*) = 20:80, 40:60, and 50:50). The scaffolds were characterized on the basis of porosity, thermal, biodegradation, mechanical, and cell viability/cytocompatibility properties. The structural properties of all the hydrogel scaffolds show significant porosity. The biodegradation of “CaP/BC-PVP” scaffold was evaluated following hydrolytic degradation. Weight loss profile, pH change, scanning electron microscopy (SEM), and Fourier Transform Infrared Spectroscopy (FTIR) study confirm the significant degradability of the scaffolds. It is observed that a 50:50_CaP/BC-PVP scaffold has the highest degree of degradation. On the other hand, the compressive strengths of CaP/BC-PVP hydrogel scaffolds are found between 0.21 to 0.31 MPa, which is comparable with the human trabecular bone. The cell viability study is performed with a human osteosarcoma Saos-2 cell line, where significant cell viability is observed in all the hydrogel scaffolds. This indicated their ability to facilitate cell growth and cell proliferation. Considering all these substantial properties, CaP/BC-PVP hydrogel scaffolds can be suggested for detailed investigation in the context of bone regeneration application.

## 1. Introduction

Different traumatic and pathological phenomena can cause significant bone fractures [1,2,3,4]. Studies have indicated that more than 20 million individuals have been affected annually by the loss of bone due to trauma and other associated diseases [1,5]. The cost of bone defect repair in the European Union (EU) is currently estimated at about 40 billion Euros, and it is expected to increase by up to 25% by 2025 [1,6,7]. Additionally, the devices used for orthopedic trauma treatment had a market of 5.7 billion in 2013, and has been predicted to reach about 7.2% of the entire global market by 2020 [8]. The current bone fracture fixation treatments include the application of stainless steel, titanium, and titanium alloys [9]. However, these metallic implants are non-degradable and thus need to be surgically removed from the body [8,10]. In addition, the mechanical properties of the devices have also occasionally not matched with the human bone, which affects the normal load transfer phenomenon from the implant to the healing bone [11]. Bone autografting has considered as the gold standard method, which facilitates the fracture healing through significant osteoconductivity and osteoinductivity [5]. However, the limitations of this method are also notable [11]. In this context, the application of polymeric biomaterials can be considered as a suitable alternative due to their composition, performance, and cost-effective production attributes [12]. 

Tissue engineering biomaterial has been devised using both natural and synthetic polymers [1,13]. Various synthetic polymers like polyethersulfone (PES), polycaprolactone (PCL), polyglycolide (PGA), poly (lactic-coglycolic acid) (PLGA), poly (l-lactic acid) (PLLA), poly (glycerol sebacate), and poly (propylene fumarate) were used in the development of biomaterials [1,14,15,16,17,18,19,20,21,22]. On the other hand, several natural polymers like collagen, chitosan, fibrin, silk, and fibroin have also been used [1,16,17,18,19,20,21,22,23,24,25]. Additionally, different polymer composites like hydroxyapatite (HA)-coated PLGA/poly (l-lactic acid) (PLLA), HA/collagen/chitosan, HA/methacrylate-co-acryloayl6-aminocaproic acid, and poly (ε-caprolactone)/tricalcium phosphate (PCL/TCP) were also notably utilized in bone tissue engineering applications [1,16]. 

Cellulose has been considered as an excellent polymer due to its notable mechanical properties and crystallinity (60%–80%) [26]. Bacterial cellulose (BC) is a significant biodegradable biopolymer [27]. Significant biocompatibility makes this biomaterial important for application as tissue engineering scaffold [28]. Studies indicated that different composite materials with BC like calcium phosphate, BC/chitosan, BC/collagen, BC/agarose, and BC/poly (3-hydroxybutyrate) have been used in tissue engineering applications [29,30,31,32]. 

Material properties like porosity, degradation properties, mechanical properties, and biocompatibility are the important attributes of tissue engineering implantable devices [32] like polymeric hydrogels [12,33,34,35,36,37,38,39]. A hydrogel is a polymeric structure which can retain a significant amount of water [40] and thus can be used notably in tissue engineering. Porosity and bio-degradability are important criteria for the applicability of polymeric tissue engineering scaffolds. Porosity of the polymeric scaffold will ensure that cell attachment occurs and facilitate significant vascularization inside the scaffold during regeneration [41]. On the other hand, the information regarding the ability of a scaffold material to disintegrate into its fundamental components after encountering with biological solution has provided the idea of the biodegradability of a material [42]. While the biodegradability of a tissue engineering scaffold promotes the development of an extracellular matrix [43], the controlled degradability is also an important aspect of a tissue engineering scaffold device [44]. Moreover, the cell viability and biocompatibility of the scaffold material indicates the cell′s attachment, proliferation, and differentiation [45]. 

Polymeric PVP-CMC hydrogel was previously developed at our laboratory as a combination of synthetic polymers, polyvinylpyrrolidone (PVP), and carboxymethyl cellulose (CMC) [36,46]. The hydrogel was also found to be degradable [46]. Furthermore, in our previous work, the development of a hydrogel scaffold using BC and PVP was also reported [47]. Research indicated that the biphasic calcium phosphate (prepared in a combination of HA and β-TCP in different ratios after being sintered above 700 °C) were successfully utilized in bone tissue engineering application [48]. In our work, the osteogenic bioactive calcium phosphates (CaP): hydroxyapatite (HA) and β-tri calcium phosphate (β-TCP) [49] are simply combined with a BC-PVP polymeric matrix in different concentrations (i.e., β-TCP:HA (*w*/*w*) = 10:90, 20:80, 40:60, 50:50, and 60:40) to prepare a calcium phosphate (CaP) filled BC-PVP based hydrogel scaffold (i.e., CaP/BC-PVP). Our previous work indicated the swelling and rheological behavior of different CaP filled BC based hydrogel scaffolds, where the swelling and rheological properties were found to be promising for the samples with β-TCP:HA = 20:80, 40:60, and 50:50 [50]. 

Few studies have been reported so far where the structural and functional properties (like the cytocompatibility and cell proliferation ability) of polymeric tissue engineering scaffolds like hydrogel scaffolds have been analyzed simultaneously [33,34,51,52,53,54,55]. Additionally, there is little work where the structural properties and functional properties of CaP filled natural polymer, BC and synthetic polymer, and a PVP based composite hydrogel scaffold were reported with the specific purpose of bone tissue regeneration. This article reports the structural and cell viability/cytocompatibility properties of CaP/BC-PVP hydrogel scaffolds. The test samples (20:80_CaP/BC-PVP, 40:60_ CaP/BC-PVP, and 50:50_ CaP/BC-PVP) were chosen on the basis of their swelling and rheological properties, which were noticed in hydrogel scaffolds [50]. Structural properties like porosity, thermal and degradation characteristics, mechanical behavior and cell viability/cytocompatibility are reported in regard to their applicability for bone tissue engineering. 

## 2. Experimental Section

### 2.1. Materials 

Polyvinylpyrrolidone K30 (PVP K30; molecular weight: 40,000), polyethylene glycol 3000 (PEG; average molecular weight: 2700–3300), agar, and β-tri calcium phosphate (β-TCP; molecular weight: 310.18 g/mol) were supplied by Fluka, Buchs, Switzerland. Hydroxyapatite (HA; molecular weight: 502.31 g/mol) was obtained from Sigma Aldrich (St. Louis, MO, USA). Glycerin was obtained from Lach-Ner Ltd., Neratovice, Czech Republic. 

Dulbecco′s modified Eagle′s medium (DMEM) and Fetal bovine serum (FBS) were obtained from Gibco-Invitrogen (Loughborough, Bishop Meadow Road, UK). Thiazolyl blue tetrazolium bromide (MTT), Dimethyl sulfoxide (DMSO) and Trypsin were obtained from AppliChem (Darmstadt, Germany). The antibiotics (Penicillin and Streptomycin) for cell cultures were from Lonza (Verviers, Belgium). Ethylenediaminetetraacetic acid (EDTA), and all other chemicals of the highest purity commercially available were purchased from local agents and distributors. All sterile plastic ware and syringe filters were from Orange Scientific (Braine-l0 Alleud, Belgium). 

### 2.2. CaP/BC-PVP Hydrogel Preparation

#### 2.2.1. Microbial Synthesis and Preparation of Homogenous Suspension of BC 

BC (holding 99% H_2_O) was synthesized in the presence of basal synthetic Hestrin-Schramm (HS) nutritive medium (pH 7.0) using *Gluconacetobacter xylinus CCM 3611^T^* (syn. *Acetobacter xylinum*) at 30°C for 15 days. 100 mL bacteriological culture bottles were inoculated with 5 mL of H.S. medium containing 96 × 10^8^ cells/mL bacteria (bacteria counted at 550 nm wavelength with Grant-Bio McFarland Densitometer DEN-1B, Grant Instruments Ltd., Shepreth, Royston, UK). The freshly prepared BC pellicle is treated with 0.5 N NaOH solution and then heated at 80 °C for 1 h to remove the possible contaminations from the BC pellicle. The newly produced BC pellicle was then treated with deionized water and the water was refreshed after 2 h of interval until the pH reached a neutral value. Thereafter, a homogenous suspension of BC (particle size: 159 nm (SD: ±11.33)) from the obtained BC mat was prepared by grinding (12 min) the BC mat in distilled water [12,31,50]. 

#### 2.2.2. Preparation of Hydrogel Scaffolds

“**BC-PVP”** hydrogel scaffold was prepared with BC (0.5 g, *w*/*v* ≈ 50 mL water based BC) suspension, where PVP (0.5 g, *w*/*v*), PEG (1 g, *w*/*v*), Agar (2 g, *w*/*v*), and glycerin (1 mL, *v*/*v*) were incorporated. The final volume of aqueous polymer solution was made to be 100 mL by adding additional distilled water [31,47]. 

The CaP filled BC-PVP based (**CaP/BC-PVP**) hydrogel scaffolds were prepared following the aforementioned composition with the addition of different amounts of CaP. The CaP was added into the “BC-PVP” polymer solution [47] in the form of β-TCP and HA, and incorporated in the following ratio: β-TCP: HA (*w*/*w*) = 20:80, 40:60, and 50:50, to achieve the calcium phosphate containing polymer solutions. 

Both the polymer solutions (with and without calcium phosphate) were then physically crosslinked by treating with moist heat and pressure. BC and PVP polymeric chains crosslinked together and formed the hydrogel′s network structure. Afterwards, 100 mL of polymer solution was treated in 250 mL sealed glass bottles under 15 lbs (107 KPa) of pressure and a temperature of 120 °C for 20 min [31,46]. Then, 25 mL of hot polymer solutions (from each sealed glass bottles) were poured into 75 mm diameter petri-dishes following the solvent casting method and allowed to cool at room temperature (22–25 °C) to obtain hydrogel scaffolds. Finally, smooth, round shaped, off white color hydrogel scaffolds were obtained. Here, while PEG and agar both had a role in the development of the polymeric hydrogel through cross-linking with base polymers like BC and PVP, the major role of PEG in calcium filled hydrogel scaffolds was to act as an agent which lowers the cellular cytotoxicity and reduces the tissue damage, while Agar acted as a notable gelling agent which maintains the hydrogel structure after solvent casting. Additionally, Glycerin acts as a humectant that retains the moisture content of the hydrogels to maintain its structural integrity [31]. 

The developed calcium filled BC-PVP based hydrogel scaffolds (Diameter: 75 mm; Thickness: 5.9–6.2 mm) are termed as “**20:80_CaP/BC-PVP**” (β-TCP:HA = 20:80), **“40:60_CaP/BC-PVP”** (β-TCP:HA = 40:60), and **“50:50_CaP/BC-PVP**” (β-TCP:HA = 50:50), respectively (as mentioned in Table 1). **BC-PVP** based hydrogel scaffold (Diameter: 75 mm; Thickness: 6.0–6.3 mm) was marked as the control.

Finally, the **BC-PVP** and **CaP/BC-PVP** hydrogel scaffolds were freeze dried or lyophilized using Scanvac Cool Safe 110-4 PRO, Lynge; lyophilizator at −80 °C temperature and 0–5 kPa pressure, and then stored for further analysis. The thickness of freeze dried samples was as follows: BC-PVP (~5.91 mm) and CaP/BC-PVP (~4.70–5.03 mm). 

### 2.3. Porosity and Void Fraction Study

The porosity of the freeze dried CaP/BC-PVP hydrogel scaffold samples was analyzed by the liquid displacement method. Three test samples (*L* = 10 mm × *B* = 10 mm × *H* = 1.5–2.0 mm = 150–200 mm^3^) for each were immersed into absolute ethanol for 48 h and examined carefully until they reached the saturation point [12]. The porosity of the samples was calculated by the following equation:

P = (*W*_2_ – *W*_1_)/*ρV*_1_ (1)

where *W*_1_ and *W*_2_ are the weight of the hydrogel scaffolds before and after the immersion into absolute ethanol, *V*_1_ is the volume of the scaffolds before immersion into ethanol, and *ρ* is the constant of the density of ethanol. During the porosity study, the test sample “BC-PVP” was kept as the control and “20:80_CaP/BC-PVP” was kept as the reference sample [12]

The void fraction of CaP/BC-PVP hydrogel scaffold were also determined by keeping the sample in ethanol [56]. Ethanol was selected as the solution because hydrogel is insoluble in ethanol [57]. The void fraction (*F*) was determined by the following equation:

*F* = *V*_h_/*V*_p_ (2)

where *V*_h_ is the volume of the hydrogel and *V*_p_ is the total volume of the pores. The total volume of the pores was determined by the amount of ethanol absorbed into the polymeric hydrogel [58]. BC-PVP (without CaP) was kept as the control. 

### 2.4. Differential Scanning Calorimetry

Differential scanning calorimetry (DSC) of freeze dried samples of CaP filled BC based hydrogel scaffolds (20:80_CaP/BC-PVP, 40:60_CaP/BC-PVP, and 50:50_CaP/BC-PVP) was performed to determine the thermal characteristics of the hydrogel scaffolds. 5–8 mg of the sample was taken. The DSC was measured with a differential scanning calorimeter (Mettler Toledo, Columbus, Ohio, USA) at a temperature range of −50 to 500 °C in the presence of the nitrogen gas flow rate at 50 mL/min [59]. 

### 2.5. Degradation Characterization

The degradability and the characteristics after degradation of the CaP/BC-PVP hydrogel scaffolds were measured through the following studies:

#### 2.5.1. Weight Loss Profile Study 

Degradability of CaP/BC-PVP hydrogel scaffolds was analyzed on the basis of weight loss profile due to hydrolytic degradation. Sections (Φ volume: 628.32–785.40 mm^3^) of freeze dried hydrogel scaffolds were prepared for this study. The hydrolytic degradation experiment was performed through incubation of the sample sections in 25 mL of physiological saline solution (Ringer′s solution; pH: 7.4) maintaining at 37 °C for 28 days (i.e., 4 weeks). The saline solution was changed weekly. The sample sections were removed from the solution at a specific time (i.e., after 7, 14, 21, or 28 days). The weight loss was also checked between 0–5 days (i.e., after 3 days) to understand the nature of the weight loss during the first week. They were then freeze dried after being washed by distilled water. The percentage of weight loss was evaluated according to the following equation [60]:

Weight loss (%) = ((*W*_0_ – *W*_t_)/*W*_0_) × 100 (3)

where *W*_0_ is the initial weight of the non-degraded freeze dried sample sections and *W*_t_ is the weight of the freeze dried biodegraded sample section at a specific time interval (i.e., 7, 14, 21, or 28 days), respectively.

#### 2.5.2. Gel Content Study

Changes in gel content of the hydrolytically degraded CaP/BC-PVP hydrogel samples were analyzed. The weights of the freeze dried hydrogel scaffolds were measured initially (*W*_0_) and after a specific time (7, 14, 21, or 28 days) (*W*_t_). The gel content of the scaffolds was measured through the following equation [61]:

Gel content = *W*_t_/*W*_0_ × 100. (4)

#### 2.5.3. Gel Density Study

The density of the freeze dried CaP/BC-PVP hydrogel scaffolds was studied at specific times (7, 14, 21, and 28 days) during the degradation study (in a physiological saline solution). The gel density ρ was determined by the following equation [62]:

*ρ* = *W*/π × (*d*/2)^2^ × *h* (5)

where *W* = weight of the scaffold, π = 3.14, *d* = diameter of the samples, and *h* = thickness of the samples.

#### 2.5.4. pH Change and Calcium Release Study

The pH change of the medium (i.e., a physiological saline solution in which scaffolds were immersed) was also analyzed with a pH meter (Lovobond pH meter, Amesbury, UK). Additionally, the percentage of calcium released by the CaP/BC-PVP scaffolds (i.e., 20:80_CaP/BC-PVP, 40:60_CaP/BC-PVP, and 50:50_CaP/BC-PVP) at specific times (i.e., 7, 14, 21, and 28 days) during degradation was also studied. The percentage of calcium release was studied by X-ray fluorescence (ARL Quant X, Spectrometer, Thermo Scientific, Waltham, MA, USA) in the presence of helium atmosphere. 

#### 2.5.5. Fourier Transform Infrared Spectroscopy (FTIR) Study

FTIR analysis was performed using fresh and hydrolytically degraded hydrogel scaffolds (over 7, 14, 21, or 28 days), which are calcium phosphate filled BC based hydrogel scaffolds designated as 20:80_CaP/BC-PVP, 40:60_CaP/BC-PVP, 50:50_CaP/BC-PVP. In each case, the freeze dried sample was used for FTIR analysis and performed at room temperature (20–22 °C). A small amount of samples with flat surface were selected from each freeze dried hydrogel scaffolds. Three repeats of experiments were conducted. FTIR was performed at wave number 600–4000 cm^−1^ with a uniform resolution of 2 cm^−1^. The ATR-FTIR analysis was conducted by using NICOLET 320 FTIR spectrophotometer with the “Omnic” software package.

#### 2.5.6. Energy Dispersive X-ray Fluorescence Study and Morphological Characterization by Scanning Electron Microscopy

The presence of calcium (Ca) and phosphate (P) in the CaP/BC-PVP scaffolds was determined by scanning electron microscope-energy dispersive X-ray fluorescence (EDX) study (SEM-EDX) with an accelerated voltage of 15 kV. 

The morphological characterization of CaP/BC-PVP hydrogel scaffolds before being degraded (i.e., freshly prepared sample) and being after degraded was also performed. The samples were first lyophilized using Scanvac Cool Safe 110-4 PRO, Lynge; lyophilizator at −80 °C temperature and 0–5 KPa pressure. The cross-sectional structure of the fresh hydrogel scaffold and hydrolytically degraded scaffolds after 7, 14, and 21 days were then studied and compared by scanning electron microscopy (SEM) “NOVA nanoSEM” (FEI, Hillsboro, OR, USA), operating in the secondary electron imaging mode at an accelerating voltage of 5–20 kV. The pore size and pore distribution of the hydrolytically degraded CaP/BC-PVP scaffolds were investigated and analyzed by using ImageJ/Fiji software (Version J2, NIH, Bethesda, MD, USA). 

### 2.6. Mechanical Property 

Mechanical behavior of the fresh freeze dried CaP/BC-PVP scaffolds was analyzed in compression mode. “BC-PVP” was kept as the control and “20:80_CaP/BC-PVP” was kept as the reference sample. Compressive strength of calcium phosphate filled BC based hydrogel scaffold was determined by using Testometric M350-5CT, England, UK at room temperature (20 °C) with a load cell having a full scale 300 kgf. Sections with 20 mm diameter and 2.5–3.5 mm thickness from freeze dried hydrogel samples were taken for the study. The study was done with a compression rate of 1 mm min^−1^. 

### 2.7. Cell Proliferation and Cytocompatibility Study

#### 2.7.1. Cell Culture

Human osteosarcoma cell line and Saos-2 were used as model systems in our study. The cells were obtained from the cell culture collection of the Institute of Experimental Morphology, Pathology, and Anthropology Museum—Bulgarian Academy of Sciences (IEMPAM-BAS). The cell cultures were grown in DMEM medium supplemented with 10% FBS, 100 U/mL penicillin, and 100 µg/mL streptomycin. The cultures were kept in a humidified incubator (Thermo Scientific, HEPA Class 100, Waltham, MA, USA) at 37 °C under 5% CO_2_ in air. For routine passages, the cells were detached using a mixture of 0.05% trypsin and 0.02% EDTA. The cell cultures were passaged 2–3 times per week (1:2 to 1:3 split). The experiments were performed during the exponential phase of cell growth.

#### 2.7.2. Preparation of Test Samples for Cytocompatibility /Cell Viability/Proliferation Study 

Three circular sections (volume: Φ 1.01–1.26 mm^3^) of each freeze dried sample (BC-PVP, 20:80_CaP/BC-PVP, 40:60_CaP/BC-PVP, and 50:50_CaP/BC-PVP) were taken for this study. Circular sections were placed in the 48-well cell culture plate, treated with 50 µL of 96% ethanol for 40 min at 30–32 °C until they were completely dried. Then, the prepared samples (BC-PVP, 20:80_CaP/BC-PVP, 40:60_CaP/BC-PVP, 50:50_CaP/BC-PVP) were sterilized under the exposure of UV radiation for 80–90 min (surface sterilization) before use.

Indirect and direct experiments for cell proliferation/cytocompatibility study were performed to evaluate the influence of the materials (with and without calcium filled hydrogel scaffolds) on cell viability and proliferation. The number of test samples was 4 (BC-PVP, 20:80_CaP/BC-PVP, 40:60_CaP/BC-PVP, and 50:50_CaP/BC-PVP) with 1 (Human osteosarcoma cell line; Saos-2) control, whereas the number of repeat experiments per condition was 8. 

#### 2.7.3. Indirect Experiments (IDE) for Cell Viability/Proliferation Study

For the cell viability/proliferation study, the Saos-2 cells were seeded in 96-well flat-bottomed microplates at a concentration of 7.5 × 10^3^ cells/well in fresh DMEM medium with 10% FBS and antibiotics. At the 24th hour, the culture medium from each well was removed and changed with 100 µL DMEM containing hydrogel scaffolds extract (sample extracts, obtained after 1-, 3-, 5-, and 7-day incubation periods). The percent of viable cells was determined using an MTT (3-(4,5-Dimethylthiazol-2-yl)-2,5-diphenyltetrazolium bromide) test. 

The MTT test was performed as described earlier [31]. Briefly, the cells were incubated for 3 h with MTT solution (0.3 mg MTT in 10 mL DMEM) at 37 °C under 5% CO_2_ condition. The formed blue MTT formazan was extracted with a mixture of absolute ethanol and DMSO (1:1, *v*/*v*). The quantitative analysis was performed by absorbance measurements in an automated microplate reader (Tecan, Sunrise™, Grödig, Austria) at 540/620 nm. 

#### 2.7.4. Direct Experiments (DE) for Cytocompatibility Study

For cytocompatibility study, each material was placed in bottom of a 48 well cell culture plate on the drop (10 µL) of FBS for 30 min at 30–32 °C in order to stick the sample to the surface of the well. Thereafter, 1 mL of DMEM (containing 10% FBS and antibiotics) was given to the wells (containing sterilized sample sections as well as empty wells that serve as controls). 

The cells (5 × 10^4^ cells/well) were seeded directly on the material sample placed on the bottom of a 48-well cell culture plate and incubated for 1, 3, 5, and 7 days in CO_2_ incubator at 37 °C. The number and viability of the cells were determined before seeding using an automated cell counter and the trypan blue dye exclusion technique (zero time). The cell viability was found to be >95% in all experiments performed. At the start of the experiment the cell numbers were equal in all wells/ between all different samples, and they were cultured in equal conditions. Cells were grown in wells without materials, which served as controls. The effect of the materials on cell viability and proliferation was studied by MTT test. MTT concentration corresponded to the volume of the plate. Like those discussed in Section 2.7.3, the cells here were also incubated in MTT solution at 37 °C under a 5% CO_2_ condition. The quantitative analysis was performed following the extraction of blue colored formazan. 

## 3. Statistical Analysis

The data for void fraction, porosity, biodegradation study, and cell viability study are presented as the mean ± standard error of the mean. The data of compressive strength is presented as mean ± standard deviation of the mean. Statistical differences between control and treated groups, as well as between the studied samples were assessed using one-way analysis of variance (ANOVA) followed by a suitable post-hoc test (Dunnett/Bonferonni) by using GraphPad Prism version 5.00 for Windows (San Diego, CA, USA) and MS Office 2010 (Redmond, WA, USA). 

## 4. Results and Discussion

### 4.1. Void Fraction and Porosity

**Figure 1:**
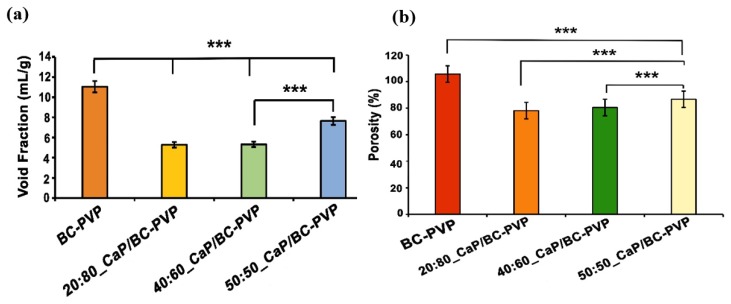
Void fraction (**a**) and Porosity (**b**) of calcium phosphate incorporated BC based hydrogel scaffolds (i.e., 20:80_CaP/BC-PVP, 40:60_CaP/BC-PVP, and 50:50_CaP/BC-PVP). Asterisk (***P < 0.0001) indicates level of statistical significance. (Statistical analysis performed through mean ± standard error of the mean and suitable post-hoc (Bonferonni test/Dunnet) test was performed followed by ANOVA). (Note: *** indicates the level of significance is explained by P value and thus written ***P < 0.0001.)

Figure 1a depicts the void fraction of CaP filled BC-PVP based hydrogel scaffolds (i.e., BC-PVP, 20:80_CaP/BC-PVP, 40:60_CaP/BC-PVP, and 50:50_CaP/BC-PVP). BC-PVP (without CaP) scaffold was kept as the control. Void fraction indicates the water/solution uptake capability of the hydrogel scaffolds. Additionally, it provides information regarding the void volume of the scaffolds. It can be seen from Figure 1a that the void fraction of BC-PVP (without CaP) is notably higher (*P* < 0.0001) than CaP/BC based scaffolds. In this study, CaPs (β-TCP/HA) are filler material, which interact with and occupy the spaces within the BC-PVP based scaffolds after application. The void fraction of 50:50_CaP/BC-PVP hydrogel scaffold is significantly higher (*P* < 0.0001) than 40:60_CaP/BC-PVP and 20:80_CaP/BC-PVP. This also indicates the higher water/solution uptake capacity of the 50:50_CaP/BC-PVP scaffold. In the filled-in polymer system there are two major types of interaction observed: one is the filler-matrix interaction and the other is the filler-filler interaction. These types of interactions strengthen the hydrogel internal structure [63]. These interactions could have the ability to affect the porous structures of the scaffold since filler-matrix interaction influences the polymeric network structures. Thus, differential loading of filler material can potentially influence the porous structures of different CaP/BC-PVP based scaffolds. Figure 1b depicts the porosity of CaP filled BC-PVP based scaffolds. BC-PVP (without CaP) and 20:80_CaP/BC-PVP scaffolds have been kept as the control. It can be seen from this figure that the porosity of CaP/BC-PVP scaffolds and BC-PVP (without CaP) was significantly different (*P* < 0.001). Additionally, the porosity of the 50:50_CaP/BC-PVP scaffold was notably higher (*P* < 0.0001) than 40:60_CaP/BC-PVP and 20:80_CaP/BC-PVP. However, the porosity of the BC-PVP based hydrogel scaffold followed the following trend: BC-PVP > 50:50_CaP/BC-PVP > 40:60_CaP/BC-PVP > 20:80_CaP/BC-PVP. Our study showed that the porosity of a material can be adjusted by changing the concentration of the structural composition/particles of that material [64]. The interaction of different concentrations of β-TCP and HA with the polymers present in the CaP/BC-PVP hydrogel scaffolds might be responsible for this trend. 

### 4.2. Differential Scanning Calorimetry

**Figure 2:**
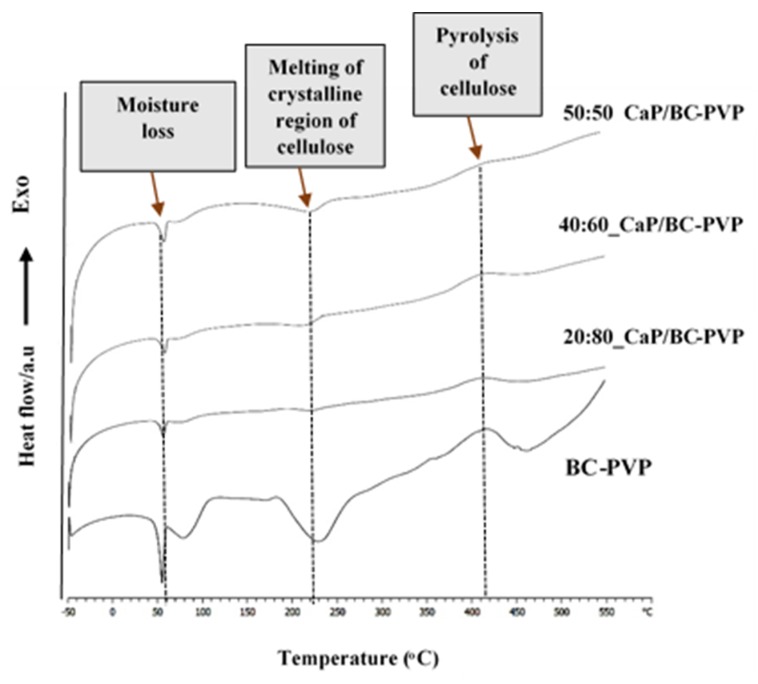
Thermal characterization of calcium phosphate incorporated BC based hydrogel scaffolds (i.e., 20:80_CaP/BC-PVP, 40:60_CaP/BC-PVP, 50:50_CaP/BC-PVP) by DSC.

Figure 2 represents the thermal characterization of CaP filled BC-PVP based hydrogel scaffolds (i.e., BC-PVP, 20:80_CaP/BC-PVP, 40:60_CaP/BC-PVP, 50:50_CaP/BC-PVP) through differential scanning calorimetry (DSC). BC-PVP (without CaP) scaffold was kept as the control. It can be seen from the figure that the DSC profiles of the hydrogel samples contained a notable endothermic peak between 50–100 °C, which occurred due to the moisture loss and which is typical for cellulose based materials [65]. This dehydration phenomenon can also be seen during a thermogravimetric analysis profile of the calcium filled hydrogel samples in our earlier study [12]. PVP polymer generally shows an endothermal peak near 100 °C, which corresponds to the moisture loss phenomenon [66]. Additionally, the endo peak occurring in the range 60–100 °C might be the melting temperature of PEG. Another endothermal event can also be observed near 250 °C, which might be due to melting of the crystalline region of cellulose [67]. The exothermic peak around 400 °C occurred due to the possible pyrolysis of cellulose. In CaP filled BC-PVP based hydrogel scaffold, the intensity of the peaks is less compared to BC-PVP (without CaP), but it indicates the significant interaction of calcium phosphate and the polymers. The intensity of the endothermic peaks gradually broadened from BC-PVP (without CaP) to 20:80_CaP/BC-PVP, 40:60_CaP/BC-PVP, 50:50_CaP/BC-PVP. The intensity of the melting of the crystalline region had a decreasing trend from 50:50 to 20:80. This might be due to the differential ratio (*w*/*w*) of CaP and its interaction with the polymers, particularly with BC. Many materials require a broad temperature range to melt [68]. The calcium phosphate melts at very high temperatures [69], and thus can be responsible for the different DSC thermogram to that of BC-PVP (without CaP). 

### 4.3. Hydrolytic Degradation Study 

#### 4.3.1. Weight Loss Profile Study

**Figure 3:**
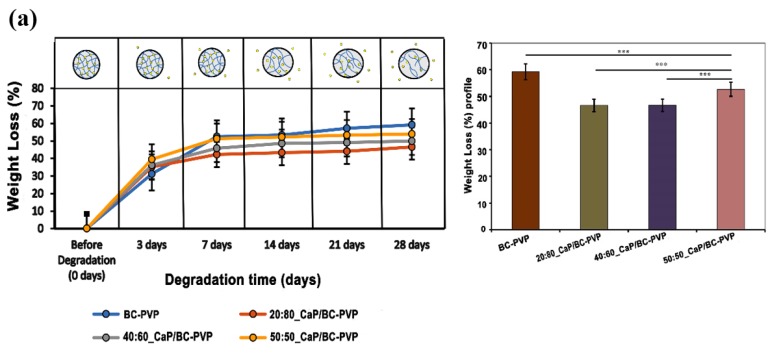

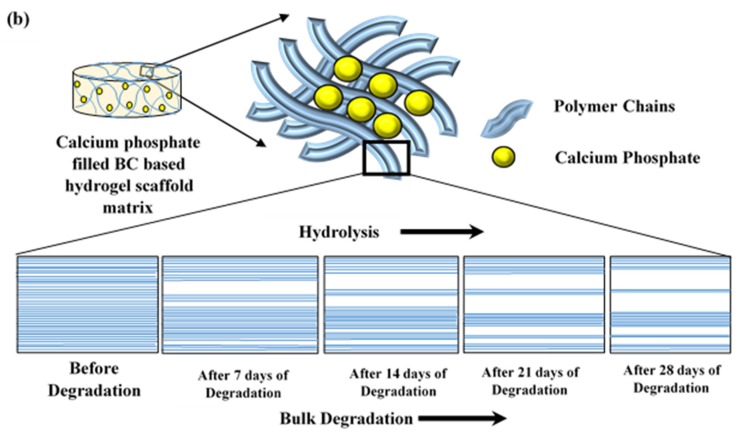
Hydrolytic degradation study of calcium phosphate filled BC based hydrogel scaffolds (i.e., 20:80_CaP/BC-PVP, 40:60_CaP/BC-PVP, and 50:50_CaP/BC-PVP). (**a**) Weight Loss (%) profiles of the scaffolds, Asterisks (***P < 0.0001) indicate the level of significance, (Statistical analysis performed through mean ± standard error of the mean and suitable post-hoc (Bonferonni test/Dunnet) test was performed followed by ANOVA). (**b**) Possible bulk degradation phenomenon of CaP filled BC based hydrogel scaffolds after 7, 14, 21, and 28 days of degradation [72].

Hydrolysis has been considered as one of the main degradation mechanisms which can occur inside the human body in the presence of the water from tissues. Research on hydrolytic degradation of biomaterials/tissue engineering scaffolds prepared with biodegradable polymers has been considered to be an important factor for biodegradation [39]. The degradation of cellulose based material is explained by the activity of cellulase enzyme. However, with the increase of time, the presence of significant cellulose amorphous structures in water based degradation medium indicates a notable chemical interaction and plausible breakdown of glycosidic bonds of cellulose [39,70,71]. Figure 3a represents the hydrolytic degradation study of CaP filled BC-PVP based hydrogel scaffolds (i.e., 20:80_CaP/BC-PVP, 40:60_CaP/BC-PVP, and 50:50_CaP/BC-PVP). BC-PVP (without CaP) scaffold was kept as a control. The figure indicates the weight loss (%) profile of CaP filled BC-PVP based hydrogel scaffolds is significant (*P* < 0.001*_n_*_=3_) after 7, 14, 21, and 28 days of hydrolytic degradation. It can be seen from the Figure 3a that, after 7 days in the physiological solution, the CaP/BC-PVP hydrogel scaffolds showed a marked 42%–52% weight loss. The weight loss between 0–5 days was also 30%–40%. However, after 14 days of hydrolytic degradation, a notable 48%–52% weight loss occurred. In particular, after 21 and 28 days, a significant weight loss was observed for 20:80_CaP/BC-PVP. The hydrogel scaffolds contain hydrophilic polymers like BC and PVP, which are also biodegradable [47]. All the BC-PVP based polymeric hydrogel scaffolds could have undergone majorly bulk degradation and surface degradation, facilitated by hydrolysis. These changes would have influenced the weight loss of the hydrogel scaffolds [72]. During the degradation period, the hydrogel scaffolds absorbed the physiological solution and swelled up. In this process, the hydrolysis was facilitated by the diffusion of water inside the matrix, which finally led to bulk degradation of the scaffolds (Figure 3b) [72]. However, the balance between the hydrolysis and the swelling characteristics of the entire gels provides the idea of the bulk degradation phenomenon of the hydrogel scaffold material [73]. Previously, the void fraction study indicated the water uptake capacity of the CaP/BC-PVP hydrogel scaffolds and showed a comparable result with the weight loss (%) profile in hydrolytic degradation. The possible significant bulk degradation phenomenon led to the weight loss of the scaffolds. BC-PVP hydrogel scaffold (i.e., the control scaffold) showed a significant degradation ability (*P* < 0.0001). On the other hand, 50:50_CaP/BC-PVP hydrogel scaffold has shown a notable degradability (*P* < 0.0001) compared to other CaP filled hydrogel scaffolds (i.e., 20:80_CaP/BC-PVP and 40:60_CaP/BC-PVP). 

It should also be noted that a CaP like hydroxyapatite has insignificant solubility in water and other physiological saline media. It maintains its mechanical property in the medium. Research showed that the ceramic scaffolds with different HA/β-TCP compositions were non-degradable in water based media, even after 1 month [42,74]. In this study, different concentrations of HA/β-TCP containing BC-PVP based hydrogel scaffolds (i.e., 20:80_CaP/BC-PVP, 40:60_CaP/BC-PVP, and 50:50_CaP/BC-PVP) have demonstrated the significant weight loss due to the hydrolytic degradation of the compositional polymers like BC and PVP. On the other hand, the poor interaction between polymer matrix and filler material could facilitate the degradation phenomenon [75,76]. The significant degradability of 50:50_CaP/BC-PVP hydrogel scaffold might be due to its weak mechanical properties and poor filler-matrix interaction other than with the other calcium phosphate filled hydrogel scaffolds. However, in general, the CaP/BC-PVP hydrogel scaffolds demonstrated a notable degradability throughout the degradation period. 

#### 4.3.2. Gel Content Study

**Figure 4:**
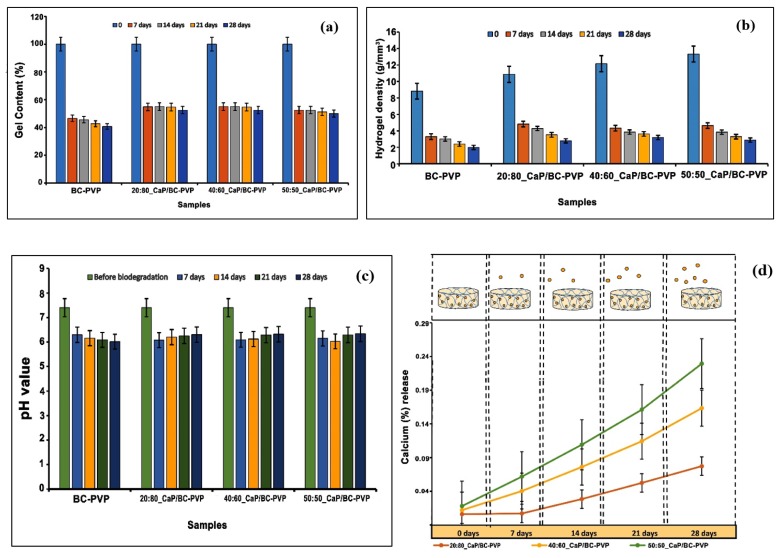
Gel content (%) histogram (**a**) and (**b**) Hydrogel density profile before and after 7, 14, 21, and 28 days of hydrolytic degradation analysis; pH change (**c**) and calcium release (%) profile (**d**) in the medium after 7, 14, 21, and 28 days of hydrolysis degradation analysis of calcium phosphate incorporated BC based hydrogel scaffolds (i.e., BC-PVP, 20:80_CaP/BC-PVP, 40:60_CaP/BC-PVP, and 50:50_CaP/BC-PVP).

The degradation of the CaP/BC-PVP hydrogels was also indicated by the change in gel content of the hydrogel scaffolds, as shown in Figure 4a. It can be observed that the gel content of the CaP filled BC based hydrogel scaffolds (i.e., 20:80_CaP/BC-PVP, 40:60_CaP/BC-PVP, and 50:50_CaP/BC-PVP) gradually decreases after hydrolytic degradation: Gel content before degradation > after 7 days > after 14 days > after 21 days > after 28 days. This might be led by the bulk degradation phenomenon caused by hydrolysis. Additionally, the change in gel content was notably seen after 28 days, which is also consistent with the weight loss profile of CaP/BC-PVP based hydrogel scaffolds. 

#### 4.3.3. Gel Density Study

Gel density indicates the structural integrity of the gel [77]. The physical changes during the degradation period involve the change in gel density [42]. Figure 4b represents the change in gel density of the CaP/BC-PVP hydrogel scaffolds due to degradation. It can be seen from the figure that the hydrogel density gradually declined with the degradation time, with the trend being gel density before degradation > after 7 days > after 14 days > after 21 days > after 28 days. This declining trend of the hydrogel density provides the necessary information of the notable gel structure which could be obtained from the bulk degradation events facilitated by significant hydrolysis [42]. On the other hand, an increasing trend of gel density before the degradation stage from BC-PVP (without CaP) to 50:50_CaP/BC-PVP hydrogel scaffold can be observed, which might be caused by the presence of different concentrations of CaP (β-TCP/HA) loading.

#### 4.3.4. pH Change and Calcium Release Study

A degradation phenomenon facilitates the physiochemical changes of the studied material, which involve the expected change of pH in the surrounding medium, facilitated by the local change of the concentration of the ions [42,78]. Figure 4c depicts the change in pH of the medium of the CaP filled BC based hydrogel scaffolds after 7, 14, 21, and 28 days of hydrolytic degradation. It can be seen that the pH of the mediums of 20:80_CaP/BC-PVP, 40:60_CaP/BC-PVP, and 50:50_CaP/BC-PVP were in decreasing order from before the degradation state (i.e., 0 days) to after 7 days of degradation, in general. However, the trend of decrease of the pH value of BC-PVP hydrogel scaffold (i.e., control) medium was the following: Before degradation > 7 days > 14 days > 21 days > 28 days. Nevertheless, the general decrease in the pH indicates the occurrence of hydrolysis inside the hydrogel structure [79], which could facilitate the bulk degradation phenomenon of the BC-PVP based scaffolds. Research demonstrated that the development of an oligomeric hydrolysis product like a carboxylic or acidic group could accelerate the decrease in the local pH, which in turn facilitates the degradation phenomenon [72]. This can be a plausible reason behind the sudden decrease in pH in the environment after 7 days of hydrolytic degradation. On the other hand, the pH values of the medium containing CaP/BC-PVP hydrogel scaffolds (20:80_CaP/BC-PVP, 40:60_CaP/BC-PVP, and 50:50_CaP/BC-PVP) are also in an increasing trend between 7 days and 28 days of hydrolytic degradation. Studies showed that the local increase of calcium ion could elevate the pH of the environment [80]. These increased values of pH of the CaP/BC-PVP hydrogel scaffold containing medium might be caused by the notable calcium release due to erosion and degradation of the CaP filled scaffolds.

Ion exchange within the study material and in the solution can result in the gradual breakdown of the structural network, which subsequently facilitates the degradation phenomenon [42]. Figure 4d represents the release profile (%) of calcium ion from the CaP filled BC based hydrogel scaffolds to the medium after 7, 14, 21, and 28 days of hydrolytic degradation. The leaching out of the filler material could also explain the degradation phenomenon of the scaffolds [81]. The release of calcium ion into the medium can be thus related with the elevated pH from 7 days to 28 days within the mediums of CaP/BC-PVP hydrogel scaffolds (Figure 4c). Interestingly, the calcium ion release profile from 50:50_CaP/BC-PVP hydrogel scaffold after 7, 14, 21, and 28 days of hydrolytic degradation was found to be higher than the 20:80_CaP/BC-PVP and 40:60_CaP/BC-PVP hydrogel scaffolds. The significant calcium release from 50:50_CaP/BC-PVP might facilitated the notable degradation of this scaffold; which is also comparable with the weight loss profile of 50:50_CaP/BC-PVP (Figure 3a). 

#### 4.3.5. Fourier Transform Infrared Spectroscopy (FTIR) Study

**Figure 5:**
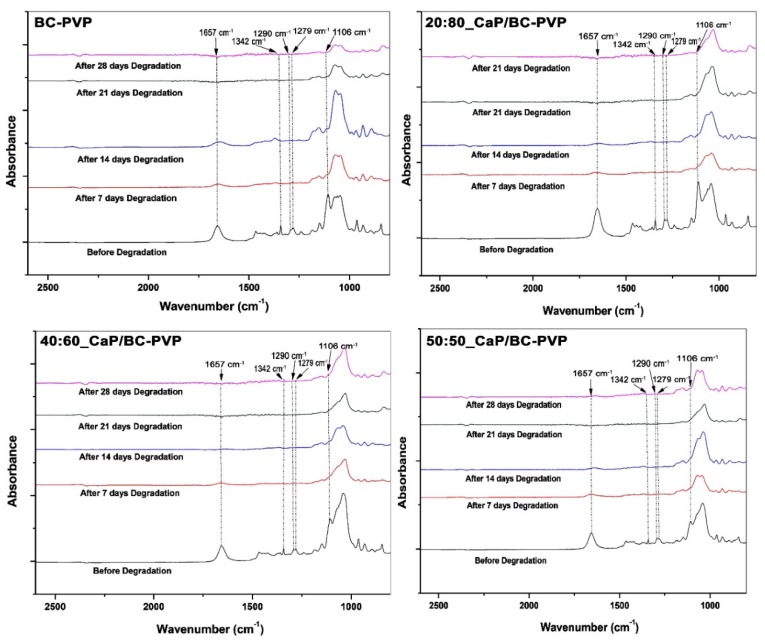
FTIR analysis of calcium phosphate incorporated BC based hydrogel scaffolds (i.e., 20:80_CaP/BC-PVP, 40:60_CaP/BC-PVP, 50:50_CaP/BC-PVP) before and after hydrolytic degradation.

Figure 5 depicts the FTIR analysis of CaP/BC-PVP hydrogel scaffolds (i.e., 20:80_CaP/BC-PVP, 40:60_CaP/BC-PVP, and 50:50_CaP/BC-PVP) before degradation and after 7, 14, 21, and 28 days of degradation. Here the data is given in the range of 800–2600 cm^−1^, as all the significant peaks and bands were revealed in this zone. BC-PVP (without CaP) was also used here as the control. The FTIR spectra provides information of the characteristic bond vibrations. It can be seen from the figure that at 1106 cm^−1^, there is a peak that corresponds to C–O stretching of glucose [82]. Additionally, the peaks at 1279 and 1342 cm^−1^ correspond to the C–H stretching and C–H_2_ deformation vibration of cellulose [83]. These peaks confirm the presence of the biopolymer, BC. On the other hand, the peaks at 1290 and 1657 cm^−1^ correspond to the C=O stretching and C-N bond vibration, which are the signature peaks of the synthetic polymer, PVP [12,77]. In addition, the peak at 1030–1043 cm^−1^ indicates the presence of PO_4_ of the CaP filled BC based hydrogel scaffolds [84]. This peak represents the presence of CaP in the hydrogel scaffold materials. CaP is a filler material which interacts with the polymer chains of the hydrogel scaffolds. As a filler, it also influences the mechanical property and at the same time it also has a role in chemical characteristics of the material. The significance presence of the CaP in the hydrogel scaffolds supports the fact that the CaP/BC-PVP based hydrogel scaffolds has the necessary ability to interact with the bone specific cells and can thereby initiate or facilitate cell proliferation. 

Research indicated that the degradation time for calcium phosphate is very long [85]. This study was done for 28 days and this period is not enough for the complete degradation of calcium phosphate.

**Figure 6:**
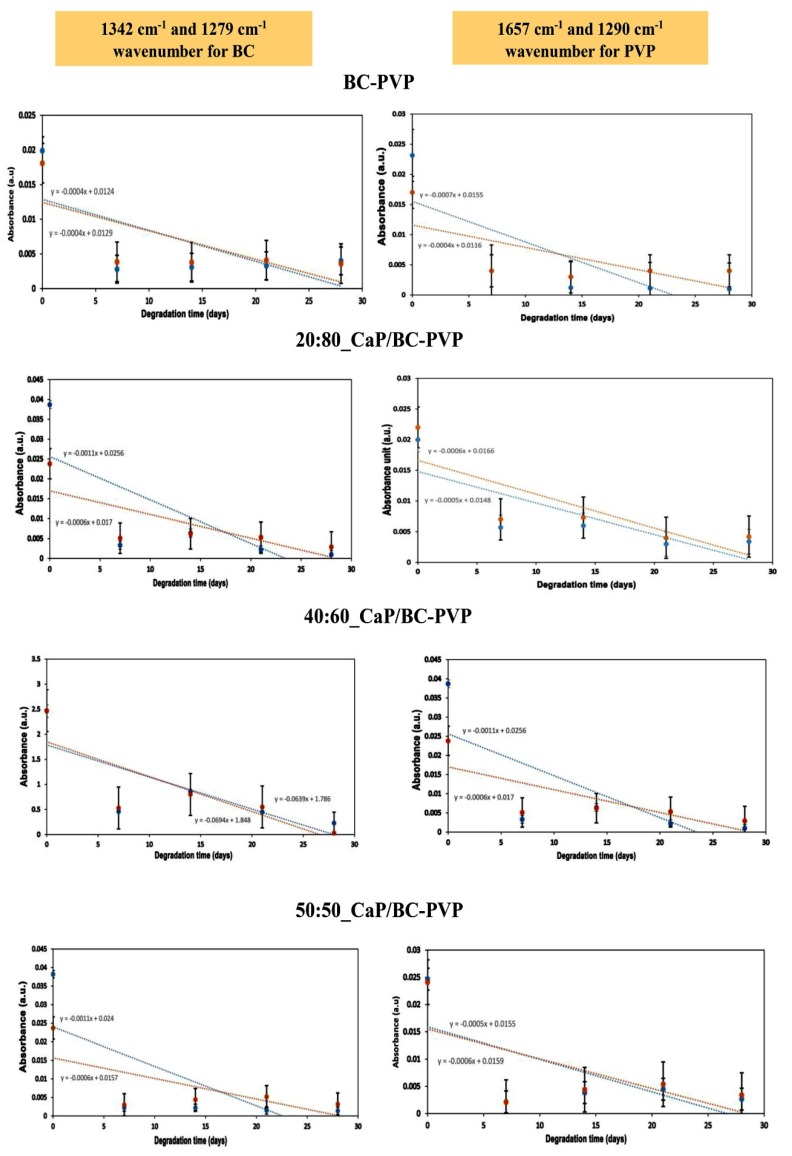
Relationship between the absorbance value of 1342, 1279, 1657, and 1290 cm^−1^ wavenumbers and hydrolytic degradation time obtained by FTIR. Trend lines showing a decreasing trend (7, 14, 21, and 28 days).

The effect of hydrolytic degradation on the CaP/BC-PVP hydrogel scaffolds can also be visualized in the Figure 5. The signature peaks of the components of the hydrogel scaffolds are in decreasing order along with increasing degradation times (i.e., before degradation > after 7 days of degradation > after 14 days of degradation > after 21 days of degradation > after 28 days of degradation). Interestingly, FTIR peak intensity is influenced by the degradation phenomenon. Degradation causes the generation of ions and compounds, which affects the peak intensity of functional groups. Thus, FTIR qualitative analysis of hydrolytic degradation is performed and shown here (Figure 6). Two signature wavenumbers of the main/major polymers BC and PVP were selected and the absorbance peaks were compared with the degradation time (i.e., after 7, 14, 21, and 28 days of degradation). The 1279 and 1342 cm^−1^ wavenumbers were selected for BC, while 1290 and 1657 cm^−1^ wavenumbers were selected for PVP. Figure 6 represents the relationship between absorbance values of the aforementioned wavenumbers and degradation time. It can be seen that a clear decreasing trend of the absorbance peaks and values occurred, which is indicated by the trend line in Figure 6. The decreasing trend of the absorbance values indicates the degradation phenomenon of the major polymeric components BC and PVP of the hydrogel scaffolds. 

#### 4.3.6. Energy Dispersive X-ray Fluorescence Study and Morphological Characterization

**Figure 7:**
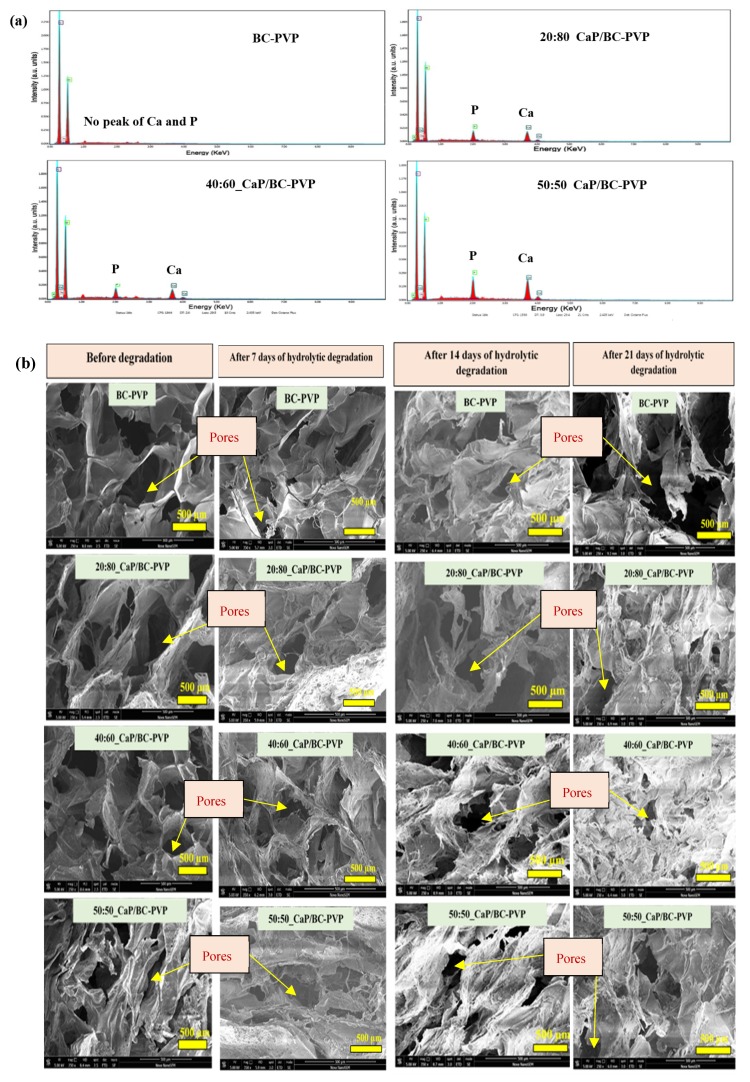

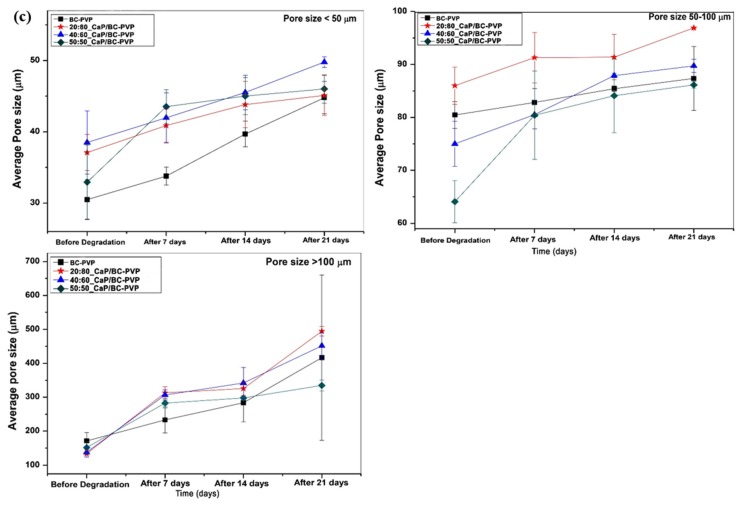
CaP/BC-PVP hydrogel scaffolds (**a**) SEM-EDX of CaP/BC-PVP hydrogel scaffolds indicates the presence of Ca and P in the scaffolds; (**b**) SEM morphology of cross-sectional structure after hydrolytic degradation; (**c**) relationship average pore size (pores > 100 µm, pores between 50–100 µm, and pores < 50 µm) with hydrolytic degradation times.

Figure 7a represents the elemental analysis through an energy dispersive X-ray (EDX) study of CaP filled BC based hydrogel scaffolds (20:80_CaP/BC-PVP, 40:60_CaP/BC-PVP, and 50:50_CaP/BC-PVP). From the EDX spectra, it can be seen that the calcium (Ca) and phosphorous (P) are present in the CaP filled BC based hydrogel scaffolds. However, only BC-PVP hydrogel scaffold showed no Ca and P. 

From the weight loss study, it was observed that the significant degradation had occurred after up to 21 days of hydrolytic degradation. Thus the morphological analysis was done after 7, 12, and 21 days of degradation. Figure 7b represents the morphology of the cross sectional structures of CaP/BC-PVP hydrogel scaffolds (20:80_CaP/BC-PVP, 40:60_CaP/BC-PVP, and 50:50_CaP/BC-PVP) before degradation and after 7, 14, and 21 days of hydrolytic degradation. A BC-PVP (without CaP) scaffold was also kept as a control. The surface structures of all CaP filled hydrogels were rough and contained much fewer notable changes during this period, and thus are not discussed further here. However, the cross-sectional structures demonstrated significant changes and are thus provided here. It can be seen from the figure that all the hydrogel scaffolds contained porous structures. The morphology of the pores between before degradation and after degradation was significant. The degraded hydrogel scaffold contained irregular pore morphologies [86]. The change in pore morphology was significant between before degradation condition and after 21 days of degradation. This might be due to a loss of interaction between the polymer chains, which causes an altered pore structure. In particular, the change in morphologies of the pores of 40:60_CaP/BC-PVP, 50:50_CaP/BC-PVP, and 20:80_CaP/BC-PVP hydrogel scaffolds are significant from before degradation to after 21 days of degradation. The loss of interaction between the polymer chains may create an possible overlapping (of the polymer phase) between each other, which can affect the morphology of the pores. Additionally, the degradation can result in the change in the matrix properties [87], which influences the morphology of the pores. The porosity and pore size have a significant relationship with the degradation rate [88]. Figure 7c indicates that the change in average pore sizes of the pores which are categorized as: >100 µm, between 50–100 µm, and < 50 µm pores. It can be seen that the average pore size of all the pores of CaP filled BC-PVP based hydrogel scaffolds (20:80_CaP/BC-PVP, 40:60_CaP/BC-PVP, 50:50_CaP/BC-PVP) are in an increasing trend with the degradation time as follows: Before degradation < After 7 days of degradation < After 14 days of degradation < After 21 days of degradation. The degradation time influences the the pore sizes in the polymer systems [89]. The hydrolytic degradation decreases the interaction of the polymer-polymer and polymer-CaP, which ultimately causes the larger pore sizes. 

### 4.4. Mechanical Property Analysis

The mechanical characteristics of fresh/before degraded freeze dried CaP filled BC-PVP based hydrogel scaffolds are represented in Table 2. “BC-PVP” was kept as the control and “20:80_CaP/BC-PVP” was kept as the reference sample. Earlier research reported that the compressive strength of human cancellous bone ranged between 0.22 and 10.44 MPa [90,91]. It can be seen from Table 2 that the compressive strength of all the BC-PVP based hydrogel scaffolds is comparable with the human trabecular or cancellous bone. Interestingly, the compressive strength of all the BC-PVP based hydrogel scaffolds can be categorized as follows: 20:80_CaP/BC-PVP > 40:60_CaP/BC-PVP > 50:50_CaP/BC-PVP > BC-PVP. A varying concentration of fillers can influence the mechanical property of the composite [45,91]. The variation of the compressive strength of CaP filled BC based hydrogel scaffolds can be the result of varying degree of polymer-filler interaction in the hydrogel composite system. The compressive strength values are given here as mean ± standard deviation (S.D). 

### 4.5. Cell Viability and Cytocompatibility Study

**Figure 8:**
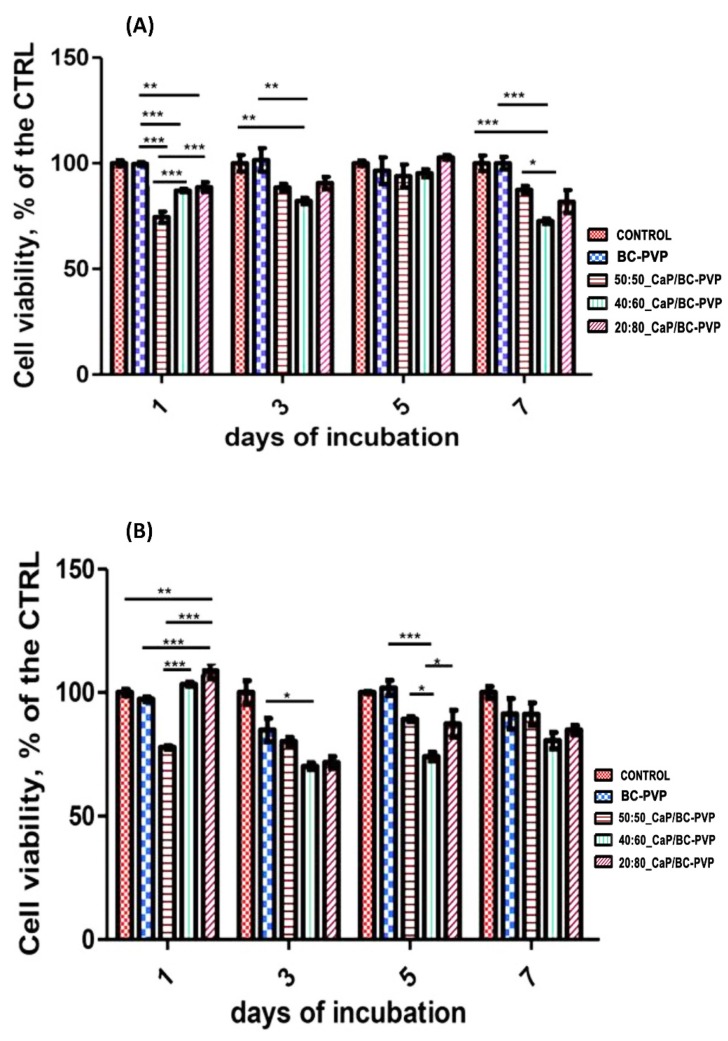
Direct and indirect cell viability for CaP/BC-PVP hydrogel scaffold with Saos-2. (**A**) Indirect experiment (IDE) and (**B**) direct experiment (DE). The asterisks indicate the level of significance (***P < 0.0001, **P < 0.001, *P < 0.05) in cell viability/proliferation.

Figure 8 indicates the cell viability/cytocompatibility property of CaP filled BC-PVP based hydrogel scaffolds (20:80_CaP/BC-PVP, 40:60_CaP/BC-PVP, 50:50_CaP/BC-PVP). It can be seen from Figure 8 that there is significant growth of cells both in DE and IDE. The cell viability of BC-PVP (without CaP) remains notable throughout 7 days in IDE. However, in DE, a sudden decrease can be seen in the percentage of growth of BC-PVP scaffold in 3 days compared to 1, 5, and 7 days of incubation. On the other hand, a gradual increase from 1 to 5 days of Saos-2 cell viability can be seen in IDE and DE for 50:50_CaP/BC-PVP. Additionally, a notable increase of cell viability can also be seen from 1 to 5 days for 40:60_CaP/BC-PVP and 20:80_CaP/BC-PVP in IDE. The cell viability was found to be more significant (*P* < 0.0001) for 40:60_CaP/BC-PVP and 20:80_CaP/BC-PVP than for 50:50_CaP/BC-PVP after 1 day of incubation at IDE. Interestingly, after 1 day of incubation, the cell viability of BC-PVP (without CaP) was found to be more significant than CaP filled hydrogels in IDE. On the other hand, the cell proliferation has found prominent (***P < 0.0001 and **P < 0.001) for 20:80_CaP/BC-PVP after 1 day of incubation in DE. This might be result from the stimulatory effect of CaP filled BC based hydrogel scaffolds. The appropriate mechanical property and calcium induced stimulatory effect might activate the cell signaling for proliferation of Saos-2 cells after 1 day of incubation [31,45]. In DE, the cell viability is also significant (*P* < 0.05) for 20:80_CaP/BC-PVP than 40:60_CaP/BC-PVP. Additionally, significant cell viability was also found for 50:50_CaP/BC-PVP (*P* < 0.05) after 5 days of incubation in DE. In this context, it should be mentioned that human Saos-2 exhibits similar characteristics to those of human osteoblast cell lines. The presence of the calcium phosphate in the medium can be notably recognized by the Saos-2. This might be the reason for the significant cell viability seen both in IDE and in DE from 1 day of incubation. Additionally, the presence of significant calcium concentration in the medium allows them to maintain proliferation signaling, which is evident from the significant cell proliferation/viability of the scaffold during 7 days of incubation. Interestingly, the BC-PVP (without CaP) scaffold shows significant cell viability in both IDE and DE. It is known that porosity and pore geometry is one of the most important factors for cell viability [45]. BC-PVP scaffold i.e., control scaffold is a significant porous scaffold [12] and is devoid of any CaP, which helps the bone specific Saos-2 cells to grow. However, from Figure 8b, it is also evident that more significant cell growth is seen after 1 day of incubation with the CaP/BC-PVP scaffolds than for the BC-PVP (without CaP) scaffold. Nevertheless, the cell proliferation for all hydrogel scaffolds is found to be promising after the 1st, 3rd, 5th, and 7th day of incubation when compared to the control, both in indirect experiment (IDE) and direct experiment (DE). 

## 5. Conclusions

This study focuses on the analysis of structural and functional properties of calcium phosphate (CaP) which incorporated bacterial cellulose (BC)-polyvinylpyrrolidone (PVP) (i.e., 20:80_CaP/BC-PVP, 40:60_CaP/BC-PVP, 50:50_CaP/BC-PVP) based hydrogel scaffolds. The evaluation of the properties of scaffolds was performed on the basis of porosity, degradability, thermal, and mechanical properties, and cell viability/cytocompatibility. The porosity of all the CaP filled BC-PVP based hydrogel scaffolds was found to be nearly 80% which is significant. DSC indicates the thermal characterization of the hydrogel scaffolds. Moreover, the weight loss profile through hydrolytic degradation confirms the significant degradability of the calcium phosphate which incorporated hydrogel scaffolds. It was determined that 50:50_CaP/BC-PVP hydrogel scaffold has the highest degree of weight loss compared to the other scaffolds. The degradation behavior was also indicated by the gel content, density studies, FTIR study, and SEM study. SEM study portrays the notable structural morphology of pores of the hydrogel scaffolds. In addition, it can be observed that average pore sizes are gradually increased with the period of hydrolytic degradation (i.e., 7, 14, and 21 days). On the other hand, compressive strengths of the hydrogel scaffolds were found to be comparable (0.21–0.31 MPa) with the human cancellous bones. In addition, significant cell viability and proliferation can be found with all the BC-PVP based hydrogel scaffolds during between 1 day and 7 days of incubation, which confirms the cytocompatibility of the CaP filled BC based hydrogel scaffolds. Thus, it can be notably concluded that the calcium phosphate incorporated polymeric hydrogel scaffolds (CaP/BC-PVP) can be utilized in bone tissue engineering applications. Further intensive in vivo investigation is recommended in animal models before their application in bone regeneration. 

## Figures and Tables

**Table 1:** Concentration (*w*/*w*) of CaP in BC-PVP based hydrogel scaffolds.

Sample Index	BC-PVP Solution (mL)	β-TCP (g)	HA (g)
BC-PVP	100	0.0	0.0
20:80_CaP/BC-PVP	99	0.2	0.8
40:60_CaP/BC-PVP	99	0.4	0.6
50:50_CaP/BC-PVP	99	0.5	0.5

**Table 2:** The compressive strength of BC based hydrogel scaffolds.

BC-PVP Based Hydrogel Scaffolds		Compressive Strength (MPa)
BC-PVP based scaffold without CaP	BC-PVP	0.21 ± 0.02
BC-PVP based scaffold with CaP	20:80_CaP/BC-PVP	0.31 ± 0.01
	40:60_CaP/BC-PVP	0.25 ± 0.04
	50:50_CaP/BC-PVP	0.24 ± 0.08
